# Supplementary material for: Antioxidative Effect of Chlorella Pyrenoidosa Protein Hydrolysates and Their Application in Krill Oil-in-Water Emulsions
Source: Mar Drugs. 2022 May 25;20(6):345. doi: 10.3390/md20060345 (PMC9229356; doi:10.3390/md20060345)
Supplement: Supplementary file 1 [file marinedrugs-20-00345-s001.zip › marinedrugs-1669256-supplementary.pdf]

Supplementary data

Results

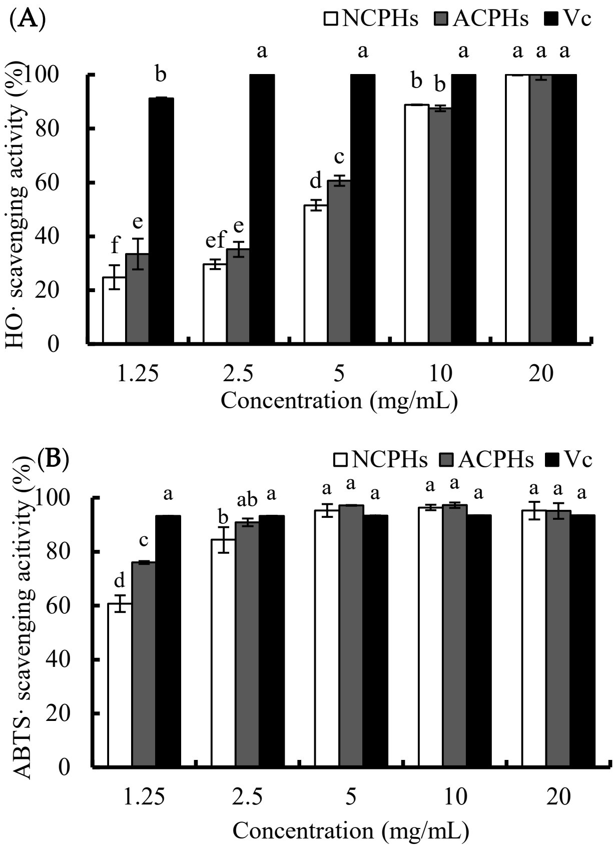

Figure S1. Hydroxyl (**A**) and ABTS (**B**) free radical scavenging activities of 0, 1.25, 2.5, 5, 10 and 20 mg/ml CPHs and VC. CPHs were obtained by neutral proteases (10000U) and alkaline protease (10000U) for 5 h, separately. Different letters above values are indicated with different significance ($p < 0.05$).
